# Digital Doppelgängers and Lifespan Extension: What Matters?

**DOI:** 10.1080/15265161.2024.2416133

**Published:** 2024-11-14

**Authors:** Samuel Iglesias, Brian D. Earp, Cristina Voinea, Sebastian Porsdam Mann, Anda Zahiu, Nancy S. Jecker, Julian Savulescu

**Affiliations:** a University of Oxford; b National University of Singapore; c University of Copenhagen; d University of Bucharest; e University of Washington

**Keywords:** Aging, digital duplicates, digital twins, life extension, longevity research, artificial intelligence

## Abstract

There is an ongoing debate about the ethics of research on lifespan extension: roughly, using medical technologies to extend biological human lives beyond the current “natural” limit of about 120 years. At the same time, there is an exploding interest in the use of artificial intelligence (AI) to create “digital twins” of persons, for example by fine-tuning large language models on data specific to particular individuals. In this paper, we consider whether digital twins (or digital doppelgängers, as we refer to them) could be a path toward a kind of life extension—or more precisely, a kind of *person* extension—that does not rely on biological continuity. We discuss relevant accounts of consciousness and personal identity and argue that digital doppelgängers may at least help us achieve some of the *aims* or ostensible goods of person-span extension, even if they may not count as literal extensions of our personhood on dominant philosophical accounts. We also consider *relational* accounts of personhood and discuss how digital doppelgängers may be able to extend personhood in a relational sense, or at least secure some of the goods associated with relevant relationships. We conclude by suggesting that a research program to investigate such issues is relevant to ongoing debates about the ethics of extending the human lifespan.

## INTRODUCTION

The ethics and governance of human lifespan extension research is a topic of growing interest and debate (Davis 2018; Porter 2023; Schloendorn 2006). With limited resources available, important questions arise about how best to prioritize and allocate funding. A central tension is between investing in technologies to extend life—pushing longevity beyond the current natural ceiling of approximately 120 years—versus focusing on improving the quality of life within a typical human lifespan (Lucke & Hall 2006).

To address such tradeoffs, however, it is necessary first to have a clear understanding of what exactly it is that proponents of life extension are attempting to prolong. In this essay, we suggest that “life”-extension may be a misnomer: it is not ultimately the prolongation of one’s *life* (i.e., biological functioning) that proponents of life extension are typically interested in. Rather, we suggest, it is the extension of one’s *self—*an experiencing being with a particular identity, life projects, social relationships, and so on—that is likely the real object of life extension projects; and it may be that human selves are only *contingently* biologically embodied.

For example, although highly speculative, it may one day be possible to upload all relevant functioning elements of one’s central nervous system onto a computer chip and to extend one’s conscious experience and sense of self in a digital form. One would then, on some views, be able to extend one’s *person-span*, meaning, roughly, one’s mental life, without necessarily also extending one’s physical or biological lifespan.

However, this possibility, too, is highly contentious and will likely not be feasible any time soon, if ever. So, it may be worth digging even deeper and asking why it is that someone might want to extend what we are calling their *person-span*. There are various potential reasons why individuals might desire to do this, and we could not hope to give an exhaustive typology. Instead, to keep our analysis focused, we will consider three main reasons:

Aim 1. **Experiential:** To continue to have subjective conscious experiences, especially when these are positive or meaningful.
Aim 2. **Legacy/Impact:** To make a lasting contribution, to have the value of one’s life and its impacts recognized by others, and to shape the future. This motivation is not about one’s own subjective experience but rather concerns one’s posthumous impact on society, communities, or the world.
Aim 3. **Relational:** To support the continued well-being of particular others and the development of particular relationships: chiefly, those one especially cares about, such as loved ones. Here the motivation is not directed at society, communities, or the world but is rather about benefiting specific individuals that one was close to in various ways.

With current technology, ensuring all three potential aims of person-span extension (preserving subjective experience, leaving a lasting legacy or having an impact on the world, and maintaining valued relationships) requires advances in biological life extension that we have suggested are unlikely in the near term. In this paper, therefore, we focus on a different means of achieving—if not the extension of our actual persons, in a thick philosophical sense—at least some of the underlying *aims* we have suggested likely constitute much of the perceived point of doing so. In particular, we explore how the second and third aims, regarding one’s legacy and relationships, might be pursued through the creation of a *digital doppelgänger*.

Although various methods might be used to create such a doppelgänger, whether now or in the future, we will be focusing on a current, highly salient example: that of using a so-called personalized large language model (LLM). This is a type of artificial intelligence, typically in the form of a chatbot, that has been carefully fine-tuned on a large amount of person-specific data, enabling it to “converse” in a style and with content that is strikingly similar to that of the person themselves (Porsdam Mann et al. 2023; see also Danaher and Nyholm 2024a, 2024b). If executed successfully, such a chatbot would be able to pass a so-called “Personalized Turing Test” (see Steinhart 2007; Caon 2020): that is, if presented with linguistic evidence only, it would not be possible to tell, beyond chance, whether you were talking to the individual or their digital “twin.”

To be clear, we will not be defending the view that a digital doppelgänger—whether LLM-based or otherwise—would necessarily amount to a form of person-span extension. Whether it would, or could, depends in part on one’s philosophical theory of personhood, and we will remain agnostic as to which theory is correct or best justified. Instead, we are suggesting that a digital doppelgänger could potentially help to fulfill at least some of the major *aims* or secure purported *goods* of extending one’s self through time (e.g., beyond current biological limits), even if it doesn’t qualify as person-span extension in its own right.

Specifically, while most likely falling short of achieving Aim 1—namely, preserving valued *subjective experience*—digital doppelgängers could, we suggest, at least partially secure goods associated with Aims 2 and 3: those associated with one’s life projects, legacy, or impact, and certain aspects of one’s interpersonal relationships. For example, a digital doppelgänger could continue writing the novel that a person couldn’t finish due to steadily worsening symptoms of Alzheimer’s disease prior to their death; or a person, through their digital doppelgänger, could, in a sense, continue conversations with friends and loved ones, thereby helping to maintain (certain aspects of) valued relationships (Voinea et al. 2024; Voinea et al. preprint).

As these examples already illustrate, and as we discuss further below, the precise sense in which a digital doppelgänger could be said to fulfill certain aims—such as finishing a novel or continuing a conversation—is not straightforward. Human persons have agency, intention, imagination, and (with various regrettable exceptions) a concern for truth and meaning. LLMs, by contrast, have none of these, or so it has been strongly alleged (Hicks et al. 2024). Accordingly, when we say that a human author has *written a novel*, to pursue that example, we typically don’t mean that she—zombie-like—typed out a very long sequence of words that just so happened to tell a story. Rather, we understand that she *intended* the words to tell a story; that the story *communicates* certain meanings, and so on. LLM-written stories don’t do that (cf. Porsdam Mann et al. 2024).

But now suppose our author carefully trains or fine-tunes a model on all of her own previous writing, including, let’s suppose, the first 80% of that big final novel: a novel she knows—due to her recent Alzheimer’s diagnosis—she likely won’t be able to finish. Further suppose that, before she is moved into a care home, where she will spend the rest of her days and do no more writing, she prepares a detailed outline of the final 20%: how she *intends* for the novel to end. Suppose she feeds this outline into the model—as a final “prompt” just waiting to be executed—with a note saying she wishes for her spouse to run the model in the event of her passing away.

And now suppose the author dies; her spouse runs the model; the personalized LLM does its thing. Suppose it generates the final 20% of the novel—in the author’s own voice, and according to her pre-determined outline—such that the end result is something the author, if still alive and free of symptoms, *would have* endorsed as a satisfactory finish to the book.

What shall we say about that 20%? Although it would be right to conclude that the LLM itself (the author’s digital doppelgänger in this example) did not *intend* for the story to come out that way; and nor did it exercise its *own* agency or imagination to communicate any truth or meaning, it could still be the case that the *author’s* intentions, meanings, and general aims for the book have nevertheless been substantially realized. This is because she used a personalized model, trained on her own past writing—which is to say, material that plausibly embeds her previous communicative efforts, intentions, ideas, skill, creativity, imagination, and so on (Earp, Porsdam Mann, Liu, et al. in press)—plus a set of forward-looking instructions that explicitly encoded her plans and desires for the future. Insofar as these author-reflecting features of the fine-tuned model played a significant role in rendering the final 20% of the novel such that the author would hypothetically endorse it, it seems right to say that she, through her digital doppelgänger, posthumously “finished” the novel.

That is the sort of thing we mean when we talk about a digital doppelgänger fulfilling certain kinds of goals.

Of course, in our example, it might have been preferable if the author had been able to finish the novel, as it were, all by herself. That would have been the *best* way of securing the good of writing that one last novel. But with the ravages of Alzheimer’s and the author’s untimely death, the best way was not an option. So, we suggest, *even if* one maintains that the author’s digital doppelgänger is in no way a literal extension of her personhood, it might still seem fair to say that it has helped to secure—albeit, in a second-best way—the ultimate good of finishing the book.

We think this sort of reasoning should apply to life-extension projects as well. We should consider, in the face of limited resources, how digital doppelgängers might serve as an alternative—possibly inferior but still valuable—means of fulfilling at least some of the aims we have suggested are likely motivating the quest for a longer lifespan (or person-span, as we would have it). For example, it could be that investing in technological and ethical research into digital doppelgängers should be given greater priority by governments than is currently the case, at least among the ones that are currently investing in life-extension projects (assuming that such expenditures are justified, which may or may not be the case; we will not evaluate that broader issue here).

To make this argument, we begin by introducing digital doppelgängers and recent advances in generative AI, especially large language models (LLMs), as well as the growing subfield of personalized LLMs. We then turn to the philosophical literature on consciousness and personal identity to evaluate whether or in what sense a digital doppelgänger could be considered an extension of a person, or—falling short of that—at least a means of achieving some of the *aims* that are typically associated with desires for such extension. We then consider some drawbacks and potential complications before drawing the essay to a close.

## WHAT ARE DIGITAL DOPPELGÄNGERS?

The term “doppelgänger” (from German *doppelgänger,* literally: double walker) is usually used to refer to a biologically unrelated person who looks and/or acts very similar to another person. A digital doppelgänger, as we use the term, is an AI program built to mimic as best possible the personality, idiosyncratic characteristics (including speaking or writing style), decisions, moral judgments, memory recall, and other linguistically encodable behavior of a given individual. We say “linguistic” behavior because, although it may soon be possible to embed digital doppelgängers into convincingly lifelike robots with synthetic physical features and movements resembling those of the person being mimicked, the relevant technology for doing so remains at an early stage of development. By contrast, a purely software-based chatbot-style doppelgänger is already possible to build, both in writing-only formats and in audio formats that incorporate vocal mimicry (Masood et al. 2023). It is also possible to create a convincing “deepfake” of a person’s face that could be shown on a computer screen and made to sync with their speech patterns, making it seem as though you were talking to the person on a video call (Masood et al. 2023); however, fully embodied robot doppelgängers are not quite there (see Sterri and Earp 2023 for discussion).

The idea of a digital doppelgänger is increasingly capturing the public’s imagination through artistic portrayals in novels such as Ishiguro’s *Klara and the Sun* and popular narratives such as HBO’s *Westworld* and Netflix’s *Black Mirror*. Real-world examples are also emerging. For example, an app called *HereAfterAI* conducts lengthy interviews with users and prompts them to recollect stories to construct a digital replica that can reflect their beliefs, desires, and memories (Morris & Brubaker 2024). Another, called Replika, specializes in virtual representations of people (Replika 2024). Perhaps most directly relevant for present purposes, YOV (You, Only Virtual) offers a service that can help one to “continue to share precious moments with a loved one, even after physical death” (YOV 2024).

Andrew Kaplan, an early adopter of HereAfterAI, age 78, wanted to be one of the first “digital humans,” stating, “[t]his is about history for me, a kind of limited immortality that creates an intimate personal experience for my future relatives who want to know where they came from” (quoted in Holley 2019). Replika’s founder, Eugenia Kuyda, initially started the company after her friend, Roman Mazurenko, was killed in a hit-and-run accident. She noted that the responses from Mazurenko’s digital doppelgänger created by the company’s technology were “spot on,” and “to be able to get back to him, to continue to have the communication we had before, it was sort of therapeutic” (quoted in Chesler 2022). James Vlahos says that he regularly interacts with the digital doppelgänger of his late father, dubbed “Dadbot”: “I do love that he can feel more present with me, with aspects of his personality that I love so much” (quoted in Chesler 2022).

While the concept itself is not limited to any one means of implementation, in this paper we focus specifically on digital doppelgängers based on personalized large language models (LLMs). LLMs are a type of generative artificial intelligence that uses a combination of deep learning, a transformer architecture, and pre-training on large volumes of human natural language data to predict the next token (a short word or a fragment of a longer word) in a sequence of tokens (sentences, paragraphs, etc.). Because they are trained on a significant portion of all written human text, state-of-the-art LLMs such as Anthropic’s Claude 3, OpenAI’s GPT-4, as well as open-source models such as LLaMA 2 show a high level of general performance across a variety of tasks (Bubeck et al. 2023).

While highly capable, these general models are often outperformed by models adapted to particular tasks via a process known as “fine-tuning” (Bucher & Martini 2024). This involves exposing a general model to a more specific dataset such that it retains its general statistical model of human language patterns (learned in its original training on general datasets) but produces outputs that are also influenced by the patterns in the more specific dataset. For example, fine-tuned models can be used to help doctors parse clinical notes more accurately than general models can (Vaid et al. 2023). Another approach to adapting general models, known as “retrieval-augmented generation” (Lewis et al. 2020), is to provide them with access to a specialized knowledge base, such as a set of PDF files, so that they can draw directly on the information contained in these external knowledge databases in composing their answers to queries.

Recently, discussion has grown around a particular type of such customized LLMs: *personalized* LLMs (Zohny et al. 2024). These are LLMs fine-tuned on, and/or provided with knowledge bases pertaining to, text written by or about a specific individual (including speech-to-text transcripts of recorded verbal output). Such models have been shown to produce answers to questions (Schwitzgebel et al. 2024) and academic prose (Porsdam Mann et al. 2023) in the style of the authors on whose articles and books they were trained. Personalized LLMs could be used for a wide variety of purposes, including the prediction of medical treatment preferences in cases where an incapacitated individual has not written an advanced directive (Earp, Porsdam Mann, Allen, et al. 2024).

Here, we argue that the ability of personalized LLMs to convincingly imitate the responses of individuals (albeit currently with varying reliability) raises intriguing possibilities for the creation of LLM-based digital doppelgängers. By fine-tuning and retrieval-augmenting an LLM based on all appropriately authorized text produced by, and/or describing, a specific individual, a model capable of responding in ways similar to that individual could be created (Danaher and Nyholm 2024a, 2024b). Depending on the quality and comprehensiveness of the training data and the underlying model, such an LLM-based digital doppelgänger could produce novel outputs in ways that reflect that individual’s knowledge, personality, and unique patterns of thought and expression.

Before proceeding, however, we should acknowledge a salient concern. It might be objected that LLMs are ill-suited for our purposes because they tend to produce grammatical but factually or logically dubious outputs, a phenomenon known as “confabulation” (sometimes also known as *hallucination*). For instance, court filings have recently been found to contain “bogus judicial decisions with bogus quotes and bogus internal citations” as lawyers have carelessly relied on tools like ChatGPT to help author them (Armstrong 2023). In another example, several top LLMs were discovered to incorrectly report that the word “strawberry” only contains two r’s (Eaton 2024). As the thought might go, if LLMs cannot be relied upon to accurately represent judicial decisions, or even the spelling of simple words, why should anyone think they would be able to accurately represent—or even convincingly mimic—something as complex and multi-faceted as a person?

We have three quick replies to this concern. First, insofar as this is a significant worry, it may only be so temporarily. It is likely that LLMs will continue their rapid rate of improvement through a combination of expanded model sizes and confabulation-mitigation techniques: the well-resourced companies behind LLMs have a strong incentive to deal with the most notorious problems plaguing their central offering. Thus, for instance, the rate of hallucination between GPT-3.5 (2022) and GPT-4 (2023) is estimated to have fallen by 11% (Chelli et al. 2024); and while it is possible there is an in-built limit on the extent of progress that can reasonably or affordably be made on such issues, for now, the trajectory suggests ongoing improvement.

Our second reply is that at least some degree of confabulation may not be all-things-considered undesirable. After all, we humans also sometimes confabulate, and this may reflect certain aspects of our cognitive architecture that could be worth preserving. As Sui et al. (2024) point out, LLM confabulation might be seen as mirroring “a human propensity to utilize increased narrativity as a cognitive resource for sense-making and communication,” suggesting, perhaps counter-intuitively, “that the tendency for LLMs to confabulate may be intimately associated with a positive capacity for coherent narrative-text generation” (1).^1^ Thus, although we concede that confabulation might be problematic for tasks related to factual knowledge retrieval, we leave room for the possibility that the causes of confabulation might prove to be a strength when it comes to producing compelling and novel human-like responses.

Finally, our third reply is that our main arguments do not depend, for their validity, on the current specific capacities of LLMs. Rather, we use LLM technology as a proof-of-concept, showing that it is already possible to create reasonably faithful digital doppelgängers using a particular type of artificial intelligence (AI) at today’s level of sophistication. What will be possible with the AI of tomorrow, whether LLM-based or otherwise, is a matter of speculation; hence, we have tried to pitch our arguments at a level that can accommodate a range of plausible technological developments in this space.

With that out of the way, we’ll proceed as follows. In the next section, we draw on the philosophical literature on personal identity and consciousness to evaluate the extent to which an AI-based digital doppelgänger could be said to constitute an extension of the self or personhood of an individual. We ultimately remain agnostic on that philosophical issue, but propose that at least some of the *aims* or ostensible *goods* of person-span expansion could plausibly be fulfilled in part by creating a digital doppelgänger. In making this argument, we consider the possibility that it could be rational to have *egoistic concern* for the “fate” of one’s digital doppelgänger; and we also explore whether one could have *non-egoistic*, relationship-oriented concerns about the same. More generally, to understand whether digital doppelgängers may be relevant to existing debates over human longevity and life extension, it will be necessary to get a grasp on what a digital doppelgänger is and how it stands in relation to the person it is based on.

## CONSCIOUSNESS AND PERSONAL IDENTITY

In this section, we touch on two main questions:

1. What are the morally relevant differences between a digital “mind” produced by the operations of a computer and a mind produced by the biological processes of the brain?
2. How do these differences affect our relation to, and—if any—appropriate moral concern for, our digital doppelgänger?

As we have argued, digital doppelgängers are increasingly able to mimic individual human personalities. In later sections, we will argue that this ability has important implications for the ethics of lifespan extension—or, what we see as the real object of such extension, namely person-span extension. This is because such models will be able to fulfill, in part, at least some of the major goals that such extension is plausibly intended to achieve. In the Introduction, we suggested that these goals likely include: (1) experiential goals, (2) legacy/impact-related goals, and (3) socio-relational goals.

We will argue that digital doppelgängers, at today’s level of technology (see previous section for caveats), can partially fulfill the second and third goals having to do with impact and relationships. However, they are *unlikely* to contribute—anytime soon, if ever—to fulfillment of the first proposed aim, the one related to continued subjective experience.

This is because LLMs, by their nature, whether personalized or not, are generally agreed not to be capable of subjective consciousness in a qualitative or phenomenal sense. Chalmers (2023) lays out several technical reasons for this conclusion, including most prominently that LLMs lack the elements stipulated to be necessary for consciousness under leading theories, such as unified agency (e.g., Schechter 2012) or a global workspace (Baars 2005). More fundamentally, however, although LLMs produce text which—if produced by a human—would certainly lead us to conclude it was conscious (for discussion, see Dodig-Crnkovic, 2023), they do so through sheer statistical modeling of human language—a method which does not require or imply consciousness (see, e.g., Bender et al. 2021). In any case, for the purposes of this article, we simply proceed on the assumption that LLMs—at least, current or near-future models—cannot help us toward the goal of extending subjective experience.^2^

One implication of this assumption is that LLM-based digital doppelgängers, as we conceive of them, are—and possibly only ever will be—*philosophical zombies* at best: that is, they might perfectly, or near-perfectly, outwardly exhibit humanlike behavior or signs of personality, but there would not be anything it is “like” to *be* a digital doppelgänger “on the inside” (Lloyd 2023). Now, just to be clear, whether LLMs—or indeed, philosophical zombies—might have *other* types of consciousness, such as so-called access consciousness (Block 2007; for a recent discussion, see Naccache 2018) will not be of concern to us here. What is critical for our purposes is the assumption that current and near-future LLMs (a) will entirely lack *subjective experience* and yet (b) will plausibly be able to pass a Personalized Turing Test (Steinhart 2007; Caon 2020) based on exclusively linguistic evidence.

What does this have to do with personal identity? As we see it, it is an open question whether subjective experience is a necessary requirement of being a *person*. Some, for instance, might be willing use the term “person” to describe certain beings who likely lack subjective experience, such as a human diagnosed as being in a permanently unconscious state (Bird-David and Israeli 2010). Others might feel that the possibility, at least, of subjective experience is a minimal condition of being a person (McInerney 1985). Suppose we accept the latter, stricter notion of personhood, such that digital doppelgängers would *not* be persons by virtue of their internal properties (whether they might be persons in a *relational* sense will be discussed later on). The question then arises: What sort of moral concern, if any, might we still be justified in having toward our digital doppelgänger: i.e., an entity that contains—that is, represents and behaves in accordance with—our particular beliefs, desires, memories, and personality traits, etc., notwithstanding that, *ex hypothesi*, it entirely lacks subjective consciousness and is therefore not a *person* according to relevant theories?^3^ We turn to this question next.

### Personal Identity and Prudential Concern

We want to evaluate whether digital doppelgängers can help to secure, at least in part, one or more of the aims or goods we’ve suggested are at the heart of desires to extend one’s life, and ultimately one’s *self*, through time. To do this, we must turn to theories of personal identity. These are attempts to answer the question of what makes an individual the same person over time, and thereby illuminate the criteria required for that individual’s continued existence.

One influential attempt to answer this question is that of philosopher Derek Parfit (1984). Rejecting certain commonsense accounts, Parfit argued that metaphysical *identity* is not what ultimately matters for the continued existence of an individual. Rather, what really matters according to Parfit is *Relation-R*: a set of diachronic connections or linking patterns that tie together certain important aspects of an individual at Time 1—such as their personality traits, memories, beliefs, and desires—to those of an individual at Time 2, such that it is rational for the Time 1 individual to have a *personal* investment in how things go for the individual at Time 2. Stated differently, to the extent that Relation-R holds between, say, your current self and a future individual, you have reason to “identify with” that individual: that is, to care about their wellbeing and to consider them a continuation of yourself in ways that matter to you, *even if* that future person is not, strictly speaking, identical to you in a metaphysical sense.

Parfit characterizes this special type of concern for a future (version of) “you” as “what matters” for personal identity. Our colleague Jeff McMahan calls it *egoistic concern* (McMahan 2002), and that is the terminology we will use as well. As McMahan states: “it can in principle be rational and appropriate to be egoistically concerned about a person in the future even if that person will not be oneself” (McMahan 2002, 42).

Importantly for our purposes, Parfit argued that Relation-R can be maintained even if a future person comes about through causes other than normal aging or biological survival. For example, following Parfit, we can imagine a case in which a future teleportation technology enables a person to be transported to Mars. The teleporter scans their body, destroys it, and then instantaneously recreates it, from local materials, on the storied red planet.

The person’s last memory, when they arrive on Mars, is stepping into the teleporter. By a strictly physical account, this memory is caused by the teleportation machine on Mars (drawing on data from a body scan of the individual back on Earth), which re-assembled the individual’s body and brain on Mars in such a way that this memory is now present. This is *not* a normal cause of the persistence of memories, much less persons, over time, but for Parfit it will do. Because Relation-R is maintained, the individual survives in a sense that really matters. And that relation, the argument goes, may be all that is required for us to be rational in having egoistic or prudential concern for a future person with whom we share it.

Exactly *what* aspects of a person must be captured by Relation-R for beings united by this relation to be justified in having egoistic concern for one another^4^ is a matter of debate. For example, some might think it is not just memories, say, but rather *consciously retrievable* or *subjectively experienced* memories that must link R-related beings for it to be rational for one to have egoistic concern for the other. If that is correct, then, given the stipulated inability of digital doppelgängers to have any type of subjective experience, it would not be possible for an individual to be truly R-related to their digital doppelgänger. At best, they could share with it what we will here dub “Relation-D”—namely, a pattern of linking relationships between various important aspects of a person (such as their memories, personality, beliefs, desires, and so on—just as with Relation-R), but where none of these attributes has a corresponding, subjective “what it is like” quality (Nagel 1974) in at least of one of the D-related beings (i.e., the digital doppelgänger). Moreover, we can assume that the digital doppelgänger, unlike the human person to which it is D-related, lacks certain other mental properties or capacities, such as (its own) agency, intention, and so on, as discussed in the Introduction.

In other words, we suggest that a person could be D-related with their digital doppelgänger (which would, again, *at the limit*, be similar to the relationship they would have with a philosophical zombie version of themselves), even if they couldn’t be R-related to it. And there would then be a further question about whether *Relation-D* is enough to justify egoistic concern, despite lacking what we are here assuming, *arguendo*, would be a necessary component of Relation-R, namely subjective experience.

Of course, some might think that Relation-R does not, in fact, require that the various connected aspects of two (or more) individuals must be consciously retrievable or subjectively experienced by both/all of them for the relation to hold. In that case, Relation-D might simply collapse into Relation-R, depending on which other aspects of individuals one believes must be present and connected for Relation-R to hold. But before we can consider such a possibility, we must first contend with the fact that, according to some critics or commentators on Parfit, *even Relation-R* is not enough to warrant (fully-fledged) egoistic concern between individuals who exist at different times.

This brings us back to Jeff McMahan (2002). McMahan points out that in hypothetical scenarios involving fully human, biological replicas (with whom we would definitely share Relation-R), many people’s intuitions seem to diverge from the view that we have reason to “identify with” such R-related beings—even *assuming* they have, among other things, a psychologically continues mental life including subjective experiences like ours. Consider *The Suicide Mission* (McMahan 2002, 57):

In a time of war, one has been chosen to carry out a military mission that will involve certain death. Although the operation of the Replicator is very expensive and has therefore been strictly rationed, one’s superiors have granted one the privilege of having a replica of oneself made prior to the mission. They will also allow one to choose, prior to the process of replication, whether one will go on the mission oneself or whether the replica will be sent. (Because one is a dutiful soldier, one’s replica will be dutiful as well. One knows that if ordered, he will go on the mission.)

Most people, McMahan suggests, would choose to send the replica, sacrificing it to save themselves. Suppose that is correct, and further suppose this choice reflects some normatively weighty consideration. The implication might be that the R-Relation is not, in fact, enough to rationally ground egoistic concern for one’s own replica, even a fully biological one that shares our most important psychological properties, including subjective experience. At least, it might suggest that people are *uncertain* that egoistic concern is warranted in such cases; or perhaps we should say that there is *less* reason for concern about the survival of a such a replica, even if there might still be *some* reason, as we will soon discuss. But now consider *The Nuclear Attack* (McMahan 2002, 58)*:*

One is an employee at the Pentagon, which has a Replicator capable of transmitting one’s cellular blueprint to a replicating booth in Alaska. One receives confirmation that a nuclear missile, targeted on the Pentagon, has penetrated the country’s defenses and will obliterate the entire area within a minute. That is just enough time to have oneself scanned and for the data to be transmitted to Alaska.

“Most of us believe,” writes McMahan, “that it would be better to have a replica created in Alaska than to be obliterated without leaving a replica” (McMahan 2002, 58). Again, suppose that’s right and that this reflects some normatively weighty consideration.

At first glance, there might seem to be some dissonance between *The Suicide Missi*on and the *The Nuclear Attack* cases. In *The Suicide Mission*, we seemed to have a reason to prefer that harm befall our R-related replica than ourselves, suggesting that Parfit’s Relation-R is insufficient grounds for egoistic concern—or at least, as we have hinted at, insufficient grounds for *confident* or *fully-fledged* egoistic concern that is equivalent to the concern we have for our future, physically-and-mentally continuous selves. In the case of *The Nuclear Attack*, however, out of an apparent egoistic concern, we nonetheless will take the continued existence of our psychological state—as instantiated in a physical replica—over its obliteration.

To address this tension, McMahan proposes a revision to Parfit’s original account of “what matters” in persisting as an individual through time (McMahan 2002, 48). In McMahan’s revision, there is a renewed emphasis on the *physical* continuity of the biologically-instantiated mental states: that is, the mental states encoding all the beliefs, desires, and memories (etc.) of an individual that are said to constitute the basis for egoistic concern. Call this stronger, physical-and-mental continuity relation the *B-Relation* due to its focus on biological continuity as the necessary substrate of the relevant psychological states. McMahan proposes that the intuition that one would prefer the pain or suffering of an R-related replica to that of themselves is grounded in the fact that replicas, despite sharing various important psychological features with us, are (crudely speaking) nonetheless different bodies and different subjects of experience.^5^

However, we shouldn’t be so quick to reject Parfit’s original account according to which Relation-R—which, unlike Relation-B, does *not* require physical continuity of the brain for egoistic concern to be rational—captures “what matters” in individual persistence. Recall that, in *The Nuclear Attack*, although it might not seem rational to have *complete* or *fully-fledged* egoistic concern for our R-related replica in Alaska, it might still seem intuitive to have *at least some* egoistic concern for it: enough, that is, for us to “identify” with our R-related replica *at least to some extent*. Enough, say, to make it egoistically reasonable to get ourselves scanned—so as to create the replica—before the missile arrives.

Some readers may get off the train there, perhaps preferring McMahan’s stricter Relation-B as the sole rational basis for egoistic or prudential concern. However, for those who are still with us, please now suppose that the Replicator in *Nuclear Attack* isn’t working. Unfortunately, there is no way to create an R-related being. As a back-up option, however, there is another machine—called a Duplicator—that can quickly build an advanced digital doppelgänger of you: the “how” is less important, but let’s just imagine that it works by fine-tuning a specially-designed, high-tech LLM on everything you have ever written or said—in text messages, emails, recorded conversations, YouTube videos, and so on, as long as you give valid authorization. Assuming that this doppelgänger would share a perfect Relation-D with you, would you have a reason to create it?

Before answering, let us add one detail. For purposes of this thought experiment, we want you to assume that the Duplicator is technologically and ethically sophisticated enough to organize all this data so that private things stay private: it won’t, for instance, allow your digital doppelgänger to share intimate text messages previously sent to your spouse with your students, boss, or children (Voinea et al. preprint). However, it could still use these messages to generate similar intimate texts under appropriate conditions: say, if and only if it can verify it is your partner who is conversing with it, among other ethical (and socio-relational/contextual) restrictions.

Returning to the question of whether survival via the Duplicator is equivalent to the other forms of survival we are discussing, suppose that in the final seconds before the nuclear missile explodes, you have a choice to press, or not to press, the fingerprint-enabled button that automatically authorizes the Duplicator to create a D-related replica of you: your perfect digital doppelgänger. In a later section, we will consider potential *non-egoistic* reasons to press the button, but for now, we are asking whether there could be any reason for *you* to want such a replica to be built—as it were, for your own sake—and if so, whether you could rationally “identify” with it *to any extent*. In other words, we want you to consider whether it would make sense for you to *at least somewhat* egoistically care about what happened either to or with it.

We assume different readers will have different intuitions here, and that some will answer firmly with a “No.” Others might strongly wish that the Replicator had been working, in this thought experiment, but still be grateful for the Duplicator as a back-up. And still others might be disinclined to use either type of machine—perhaps believing that, if they were to use it, whatever came out the other end would simply not be a fitting object of egoistic concern, full stop.

We are not certain about this question ourselves. But we think it might be reasonable, at this point, to propose a range of potential egoistic concerns roughly grouped as shown in Table 1.

**Table 1.** A proposed range of justifications for egoistic concern.

*B-related beings.* We have, perhaps, the strongest reason to be egoistically concerned about future entities that hold B-relations with us, namely in the functional continuity of the brain in producing consciousness. This egoistic concern can be diminished, but not eliminated, as the entity loses psychological continuity with us. *R-related beings.* We may have the second strongest reason to be egoistically concerned with conscious entities that hold Relation-R with us, in proportion to their strength of psychological continuity with us. This reason could have to do, for instance, with their ability to further our legacy, which may hold some subjective and objective conditions of satisfaction (see later discussion). *D-related beings.* We may have third strongest reason to be egoistically concerned with D-related entities—i.e., entities that lack subjective experience of any kind, yet which *simulate* Relation-R with us—in proportion to the strength of their continuity with us in terms of outward linguistic behavior. As they lack subjective consciousness, egoistic concern grounded in this relation would only be rationalized by their ability to further aims that have objective conditions of satisfaction (see later discussion).

One might object that this account is mis-ordered and that psychological continuity is a stronger criterion for egoistic concern than functional continuity of the brain. Or one might object that there are no cases in which one must consider a tradeoff between a future B-related individual and a different, future R-related individual, as replications of either type are fiction. What we want to emphasize here is that all three relations—R, B, and D—*admit of degrees*. Accordingly, whatever one’s preferred account of personal identity, there may exist a point at which sufficiently-diminished continuity in B-relations (functional continuity of the brain) or R-relations (subjectively experienced psychological continuity) could become superseded as a basis for egoistic concern by the strength of one’s connection to a future D-related entity, *even if* that entity is in no sense a person.

Consider, for example, our writer from the beginning whose ambitions are frustrated by an Alzheimer’s diagnosis. Might she rationally choose to spend her fleeting lucid months training a digital doppelgänger rather than rushing out her long-delayed novel? How financial resources are allocated might be similarly reevaluated, as both palliative care and the development and maintenance of her doppelgänger have costs. She might evaluate her doppelgänger to have a very high likelihood of completing her work in a manner she would deem acceptable (strong Relation-D) and her immediate future self to have a very low likelihood of doing so (weak Relation-R or Relation-B). In this case we might say her choice to develop the doppelgänger is egoistically rational.

Alternatively, she might, say, evaluate her novel’s being finished to be relatively less important than traveling the world with her family, and for that reason disfavor creating the doppelgänger. There are various other tradeoffs that could be imagined. The point is, several factors—including the degree to which we relate to our future biologically- or psychologically-continuous selves, the degree to which we relate to our digital doppelgänger, and the relative importance of certain kinds of unfinished plans—will factor critically into how we choose to spend our time and resources and manage any relevant tradeoffs. And in our view, based on the trajectory of current technological developments including the quality and quantity of data we are capturing about ourselves, the potential verisimilitude of one’s digital doppelgänger to oneself, and hence the strength of Relation-D between those two entities, will increase significantly over time.

In the following sections, we expand on how digital doppelgängers can contribute to completing our life plans and to continuing aspects of our close relationships, in ways that we have reason to value, *whether or not* we are willing to consider our digital doppelgängers as literal extensions of our selves.

## PARTLY ACHIEVING THE GOODS OF PERSON-SPAN EXTENSION

Some argue that individuals’ interests can be negatively affected after their death, for example, through reputational harm (Pitcher 1984, Feinberg 2017).^6^ But if individuals can be harmed after death, this means they can also be benefited by events occurring after their death (Boonin 2019). On an objective, non-hedonistic conception of well-being, certain posthumous events can thus contribute positively to the overall value of an individual’s life. Just as the preservation and recognition of one’s artistic or literary works can be said to enhance an individual’s well-being by ensuring that their legacy endures, so too, we propose, can the existence and influence of a digital doppelgänger continue to impact the world in ways considered positive after one’s death. (Of course, it might also impact the world in ways considered very negative; see Danaher and Nyholm 2024a for a thoughtful discussion.)

In what follows, we show how digital doppelgängers might, in some relevant sense, continue an individual’s legacy by satisfying relevant desires, or fulfilling relevant plans, that the individual had while they were alive. However, as we will also discuss, some of the potential goods of person-span extension may have a *relational* nature, rather than a strictly individualistic one: that is, their value is derived from or depends on others’ recognition, evaluation, or ongoing experience, such as those that might be had or carried out by significant others.

Legacy is an umbrella concept, encompassing both material possessions and non-tangible goods such as meaningful bonds forged during one’s lifetime, the broader impact of one’s life projects and commitments, and the network of values someone stood for, to name a few. Legacy has an interest-preserving function: it is the primary vehicle through which individuals continue to leave a mark on the world after their biological demise. The desire to leave a legacy has long been explored in terms of a “hope that we have the potential for immortality” (Birren 1988, 154), whether purely symbolic (Kotre 1984), or more substantive, along the lines we consider next.

### Legacy/Impact

When discussing hypothetical cases of person-division, such as a scenario in which each cerebral hemisphere of a person is transplanted into a new body, Parfit (1984) introduces the idea of having interests that we’ll call *non-experiential*. He suggests that each of the offshoots, call them B and C, of the original individual, A, could then carry on different cherished life projects A had. For example, B could finally finish the patchwork quilt that A always intended to finish, while C could pursue the career in science A always regretted not choosing. Other examples might include having the cathedral one has designed constructed, getting a piece of legislation passed, having one’s child’s life go well, or even winning a war. We might speak, for instance, of certain causes being *worth dying for*, where the question of whether it was, in fact, “worth it” might be seen as being contingent on facts that obtain *after* one has died. These kinds of desires (e.g., that one’s own side wins in a war) don’t need to be *experienced* to be satisfied (hence, ‘non-experiential’) and can be said instead to have *objective* conditions of satisfaction.

These stand in contrast to desires that have *subjective* conditions of satisfaction: those that must be *experienced* to be satisfied. For instance, if someone has the goal of living long enough to see Haley’s Comet in one’s old age, we might sense that something is lost if that person is in a coma during its return. Fulfilling desires with *subjective* conditions of satisfaction seems dubious if the being in question lacks subjective experience altogether; not so for desires with objective conditions of satisfaction.

Building on this insight, we suggest that, for many people, having a digital doppelgänger continue their societal influence or their distinctive projects and commitments, could have genuine egoistic value—even if that replica is not subjectively conscious and will not host a continuation of their own phenomenology. People have prudential reasons to care about what happens after their death. It makes sense to prefer that one’s efforts and life-plans, or the shaping of one’s reputation in light of their fulfillment, are not completely dissolved after one’s passing.

After all, the eventual outcomes of one’s life projects following one’s death can cast a retroactive shadow on their lifetime achievements. For example, if an individual dedicates many years to a life project that ultimately fails posthumously, their efforts might be judged as misguided or pointless, regardless of their initial potential. Posthumous events can thus influence the way we evaluate someone’s life in retrospect: whether someone led a happy, successful, fulfilled life or not (Rozin and Stellar 2009). Accordingly, it can be rational to care about what happens after we die and also to wish to continue exerting a positive influence through our legacy (Scarre 2001).

However, our potential for exerting continued influence is not limited to life projects. Certain life experiences that are defining for how individuals view themselves can also be of great significance. When people are asked about what they would ultimately like to pass on after death, they oftentimes refer to a legacy of *values* that are closely tied to aspects of their sense of identity, or to the roles and traits they perceive as constitutive of who they are (Hunter 2008, Zacher et al. 2011). Legacies of values are often especially important for people who experienced hardships or marginalization: legacies are passed on as a collection of guiding principles meant to assist others in navigating similar difficulties. For example, HIV-infected women created video legacies for their families, imparting advice and guidance to help their loved ones lead successful lives despite the challenges they might face (Barnes, Taylor-Brown, and Wiener 1997). These legacy videos were important for the women creating them as they were seen as a way to take control of the narrative they left for their offspring posthumously. This shows that one might wish that one’s life experience, considered as a token of oneself, continues to shape and inspire others. These legacies may have moral as well as prudential dimensions.

Many people prefer to make a lasting contribution, to have the value of their life and its impacts continue to be recognized by others after they are gone, and to shape the future. Such aims do not necessarily presuppose that the creator’s subjective experience continues, although they may include prior subjective preferences or feelings related to posthumous impact. Hence, a digital doppelgänger could, in some sense, continue the story of the original person’s life and extend it: by accomplishing or fulfilling that person’s goals and desires, or at least goals and desires that do not require subjective experience to be satisfied. These non-experiential interests—namely, in posthumous impact and ongoing social influence—might give us some egoistic reasons to care about what happens to, and with, our digital doppelgängers after we die.

### Relational Identity

A potential objection to what we have so far suggested is that it all seems rather, well, *egoistic.* And yet, in analyzing the ethics of research into lifespan extension, or indeed person-span extension as we are proposing here, it is necessary to consider rights and interests other than purely egoistic ones. For example, there might be worries at a societal level about the continued, albeit partial, temporal persistence of potentially limitless representations of individuals. It is one thing for a society to hold its (biologically) deceased in memory, or in finite artifacts, such as photographs or clips of video; it is another for the “deceased” to *keep on going*—potentially *en masse*—producing new ideas, having new conversations, and so on. Perhaps it is ultimately good for the living to learn how to grieve and let go.

Without presuming to offer anything like a comprehensive analysis of the society-wide ethical implications of digital doppelgängers (on that front, we recommend the recent essays by Danaher and Nyholm cited earlier), we will close with a few thoughts about *relationships*.

Recall the stories of James Vlahos, Eugenia Kuyda or Andrew Kaplan that we raised earlier in the paper. In those cases, it was not the continued subjective consciousness of the digitally simulated individual that seemed to matter the most; rather, it was a certain type of *relationship* between these persons and their loved ones that was experienced as significant. How might we begin to make sense of such testimony? Are these people adopting a plausible or reasonable attitude, or are they simply in denial, unable to process the sudden non-continuance of a person they have held so dear? Could an ongoing “relationship” between an organic human being and a digital doppelgänger of a recently deceased loved one be of any real value in itself?

We will touch on this question later in this section. First, however, we’d like to make a broader point about the concept of *relational identity*. Essentially, this approach posits that our identity cannot be isolated from the outside world, the communities of which we are a part, the norms guiding our conduct, and, more importantly, the relationships we cultivate with others across a lifespan (Andersen and Chen 2002). At times, these meaningful relationships end up being the central features of our so-called social or practical identities, so much so that certain roles such as mother, father, teacher, doctor, artist, and so on, partly constitute who we are. Thus, we are inclined to say things like, “I am a mother, father, teacher, doctor, or artist” rather than simply “I parent, teach, practice medicine, or make art.”

The case of patients in a persistent vegetative state, who might be considered neither dead nor alive, neither subjects nor objects, shows the potential for social relations to shape our status (that is, our ability to be meaningfully recognized) as persons (Bird-David and Israeli 2010). These patients challenge the traditional understanding of a person as “an individual who experiences his or her body-in-the-world, a self-aware individual who constitutes agency” (Bird-David and Israeli 2010, 55). Through everyday engagement with patients in permanent vegetative states, caretakers maintain or generate “different senses of their personhood” (Bird-David and Israeli 2010, 62). The relational perspective on personhood illuminates how even individuals who are no longer, and may never again, have subjective experiences can still be considered persons in some sense, namely, by virtue of their relationships with others.

Of course, it could be objected that these are one-sided, non-reciprocal relationships—or as some might see it, not *relationships* at all. Like the individual who, say, claims to be in love with a celebrity figure whom they have never met, and who doesn’t know of the individual’s existence, one-sided “relationships” can sometimes be unhealthy or delusional. But they can also be healthy and meaningful. Someone who reads to their unconscious relative is, perhaps, engaging in a relationship that “keeps the person alive” by connecting past with current memories, which could potentially aid in their own development or grieving. While non-reciprocal, reciprocity might not be a necessary ingredient of all meaningful relationships. Our engagement with others continually shapes us and becomes a part of who we are (or who we consider ourselves to be), just as those individuals for whom we have held significance are influenced by us (Taylor 1989; Lindemann 2016; Iftode et al. 2024; see also Velleman 2005).^7^

We propose then to expand our previous set of reasons to be concerned about one’s own future self by incorporating relational concerns, according to which the continuation of certain relational dynamics might reasonably be valued, even if one of the dyad members lacks subjective experience. Of course, as we have already alluded to, some may hold that for a relationship to be valuable, or even to count as a relationship at all, it must be the case that both parties subjectively experience the interactions between them (Turkle 2007). If that is the case, then those who report valuing their ongoing relationship with, say, the digital doppelgänger of a deceased loved one are simply mistaken—tragically seeming to be engaged in a valuable relationship, but in reality, experiencing nothing of the sort.

Alternatively, if we take a graded or hierarchical view of the status or value of relationships, it might be acknowledged that the *most* valuable, or real, relationships are those between two persons, both of whom are able to subjectively experience the world, and each other. But if we imagine a spouse who continues to love and relate to their partner who is alive, but in a coma, possibly without subjective experience of any kind, it might seem, not just cruel or unfeeling, but wrongheaded to deny that the relationship had any value, or to insist that the living spouse was making an error in experiencing it as such. A similar kind of analysis might apply to the relationship between a person and a digital doppelgänger of someone they love. Digital doppelgängers, despite currently lacking subjective experiences, can at least be a means for remembering, but also potentially sustaining, valued aspects of our relationships with others (Voinea 2024; Voinea et al. 2024; Voinea et al. preprint).

## WHAT MATTERS IN PERSON-SPAN EXPANSION

If the preceding arguments are persuasive, or at least broadly plausible, what might follow for human longevity and life extension research? As noted at the paper’s start, there are pragmatic reasons for getting clear about what matters most about life extension in light of resource constraints. Plausibly, we have argued, it is not biological “life” as such that we care most about extending, but rather, in some sense, our *selves*. And why do we care about extending our selves? We have suggested there are various reasons for this, associated with different types of goals—experiential, legacy-related, and relational—some of which can be at least partly met by the creation and use of a digital doppelgänger. Therefore, in weighing up tradeoffs in relation to resource expenditure on longevity research, it may be prudent to consider a wider range of projects that could be understood as falling under this umbrella.

Reflecting on what matters in human life extension also invites us to think about different types of things that matter, from both egoistic and non-egoistic perspectives. As we have argued, there are some *objective* occurrences (e.g., finishing a novel after we die) that one might rationally desire to have happen and also reasonably consider to be a contribution to one’s overall interests or well-being. Some of these occurrences, we’ve suggested, could be brought about by a digital doppelgänger. In other words, aims that require *subjective* experiences do not exhaust the kinds of things we care deeply about; rather, they represent just one aspect of what matters. Other kinds of values and achievements can live on after death.

Our analysis also established that we have non-egoistic reasons to want to extend our selves, tied to the relationships we have with close others: digital doppelgängers, judiciously used, could potentially satisfy some such reasons, at least in part. More generally, if we follow Parfit in viewing what matters in individual persistence as a matter of degree—rather than all-or-nothing—then even a *partial* continuation of oneself may hold value. If not in terms of McMahan’s Relation-B, then perhaps Parfit’s Relation-R. And if not Relation-R, then our Relation-D. Finally, even if one rejects the view that the last of these relations, at least, namely Relation-D, could ever be sufficient for person-span extension—that is, in any sense, or to any degree—we’ve suggested that a D-related being could still plausibly help to meet some of the aims, or bring about some of the goods, associated with such extension.

## CONCLUSION

In this essay, we have focused on ways a digital doppelgänger could advance at least some of the interests a person might aim to promote through traditional forms of biological self-extension, not only from the perspective of egoistic concern but also from the perspective of relationships. However, this assumes it would be overall *good* to advance these interests, in something like the way that proponents of lifespan extension argue for. Therefore, it must be acknowledged that some people are quite critical of lifespan extension (e.g., Callahan 1995): they think we should be satisfied with a healthy and finite life not much longer than the average lifespan now.

For this paper, we take no position on such debates; our focus has been deliberately narrow. We can be read, therefore, as making a series of conditional claims: namely, *insofar* as it is reasonable, at an individual level, to care about extending one’s biological lifespan—this may sometimes have more to do with a desire to extend one’s *self* through time, rather than one’s “life” as such. And if *that* is a desirable goal, but one that cannot readily be achieved with existing technology, or which might not be feasible for most people for a long time to come, it could still be the case that a digital doppelgänger could serve as a “second-best” option, in the sense that it could help to fulfill at least *some* of the reasons we might care about self-extension in the first place.

But whether you agree with our specific proposal about digital doppelgängers or not, our point about needing to identify the underlying reasons for seeking to extend one’s life and/or self matters for research and policy. For example, those who are considering the pros and cons of investing in human lifespan extension—including any tradeoffs for funding different initiatives—should first get clear about what it is that people fundamentally care about in living longer lives. We have suggested some possible answers here; there may be others. Either way, reflecting on these types of reasons should open up space for thinking more creatively about various other ways of potentially addressing people’s concerns, if only in part.

In closing, we wish to stress that we have left many important aspects of this topic almost entirely to one side. For example, there are numerous ethical (as well as legal and policy) concerns that can and should be raised about digital doppelgängers, some of which we hope to address in forthcoming work, and which other authors have already begun to explore (e.g., Danaher and Nyholm 2024a, 2024b). There is also much more that could be said about how, in practice, governments or other funding bodies currently invested in longevity research should allocate resources given the arguments and claims in this essay: for example, claims about the relative ease with which digital doppelgängers can be created compared to the evident difficulties of prolonging biological life. But first, it will be necessary to continue sharpening our arguments and claims in response to debate and dialogue with our peers. We hope this essay serves as an initial step toward an ongoing, fruitful conversation, and we look forward to the critiques and commentaries to follow.

## Footnotes

1 Others have argued that hallucinations in LLMs can even be understood instead as a type of “creativity” and can thus be useful in some cases (Jiang et al. 2024).

2 To be sure, this is not a settled matter, and some may be more open to the possibility of LLM consciousness in the reasonably near future (Long et al. preprint). Nevertheless, to constrain our theorizing and for the sake of clarity in discussing personal identity in the following sections, we will simply assume that something like biological naturalism is correct, according to which phenomenal consciousness and subjective experience are emergent properties of certain physical processes in the brain (Searle 2007). On this view, a computer program, no matter how accurately it simulates human behavior, would not itself generate genuine subjective experience, just as a computer simulation of bioluminescence doesn’t actually produce visible light.

3 This question is consistent with recent arguments of Jennifer Blumenthal-Barby (2024), who claims that instead of *first* having to determine whether an entity is a person to know how it is rational or moral to treat it, we can sometimes make progress on those latter questions by appealing directly to certain properties of the entity, irrespective of whether these are sufficient for personhood (see also Dranseika et al. 2024; for an alternative perspective, see Koplin et al. 2024).

4 Or at least, the “Time 1” individual having egoistic concern for the “Time 2” individual. Whether we can rationally have egoistic concern for our past selves—or for past individuals with whom we are R-related—is an interesting question we will not be getting in to. Instead, we are focused exclusively on the forward-looking case: how it is rational for us, while living, to relate to a digital doppelgänger that persists after our biological death. On the possibility and ethics of creating multiple digital doppelgängers (i.e., of oneself) while one is still alive, see Danaher and Nyholm (2024b) and Voinea et al. (2024).

5 If McMahan is right, then we should narrow the set of criteria that form the basis of egoistic concern to those that more closely align with personal identity as traditionally conceived (i.e., in biological-continuity accounts). This is referred to as the Embodied Mind Account, according to which “the criterion of personal identity is physical and minimal functional continuity of the brain,” and that “physical but not organizational continuity of the brain is necessary in order for egoistic concern to be rational” (McMahan 2002, p. 69). But we are not so sure McMahan is right, as we explain in the text.

6 Even though this intuition is common, it remains highly controversial as its normative status is unclear (see, for example, Partridge 1981; Harris 2013).

7 These considerations are not merely theoretical; they are deeply entrenched in how we relate to others. Fear of death, which is a multi-dimensional bundle of emotions, often incorporates the fear of how our demise will impact those we love (Abdel-Khalek 2002; Cicirelli 1998). One study of the causes of death anxiety found that “separation from others through death (either one’s own death or the death of another) is one of the most fearsome aspects of death, a fear that is shared by the majority of respondents” (Bath 2010). This might be because we know our departure may deprive those we care about and who care for us of an essential relationship for life. To the extent, then, that their engagement with our digital doppelgängers after we die can be a healthy part of the grieving process, there are reasons—besides egoistic ones—to wish to create them.
